# A SINE Insertion in *ATP1B2* in Belgian Shepherd Dogs Affected by Spongy Degeneration with Cerebellar Ataxia (SDCA2)

**DOI:** 10.1534/g3.117.043018

**Published:** 2017-06-15

**Authors:** Nico Mauri, Miriam Kleiter, Elisabeth Dietschi, Michael Leschnik, Sandra Högler, Michaela Wiedmer, Joëlle Dietrich, Diana Henke, Frank Steffen, Simone Schuller, Corinne Gurtner, Nadine Stokar-Regenscheit, Donal O’Toole, Thomas Bilzer, Christiane Herden, Anna Oevermann, Vidhya Jagannathan, Tosso Leeb

**Affiliations:** * Institute of Genetics, Vetsuisse Faculty, University of Bern, 3001, Switzerland; † Department for Companion Animals and Horses, University Clinic for Small Animals, University of Veterinary Medicine Vienna, 1210, Austria; ‡ Department of Pathobiology, Institute of Pathology and Forensic Veterinary Medicine, University of Veterinary Medicine Vienna, 1210, Austria; § Division of Clinical Neurology, Department of Clinical Veterinary Medicine, Vetsuisse Faculty, University of Bern, 3001, Switzerland; ** Section of Neurology, Department of Small Animals, Vetsuisse Faculty, University of Zurich, 8057, Switzerland; †† Division of Small Animal Internal Medicine, Department of Clinical Veterinary Medicine, Vetsuisse Faculty, University of Bern, 3001, Switzerland; ‡‡ Department of Infectious Diseases and Pathobiology, Institute of Animal Pathology, Vetsuisse Faculty, University of Bern, 3001, Switzerland; §§ Wyoming State Veterinary Laboratory, University of Wyoming, Laramie, Wyoming 82070; *** Institute of Neuropathology, University Hospital Düsseldorf, 40225, Germany; ††† Institute of Veterinary Pathology, Justus Liebig University Giessen, 35392, Germany; ‡‡‡ Division of Neurological Sciences, Department of Clinical Research and Veterinary Public Health, Vetsuisse Faculty, University of Bern, 3001, Switzerland

**Keywords:** *Canis familiaris*, canine, Malinois, Na^+^/K^+^-ATPase, β_2_ subunit, adhesion molecule on glia, AMOG, astrocytes, brain, central nervous system, epilepsy, KCNJ10, cerebellar dysfunction

## Abstract

Spongy degeneration with cerebellar ataxia (SDCA) is a genetically heterogeneous neurodegenerative disorder with autosomal recessive inheritance in Malinois dogs, one of the four varieties of the Belgian Shepherd breed. Using a combined linkage and homozygosity mapping approach we identified an ∼10.6 Mb critical interval on chromosome 5 in a Malinois family with four puppies affected by cerebellar dysfunction. Visual inspection of the 10.6 Mb interval in whole-genome sequencing data from one affected puppy revealed a 227 bp SINE insertion into the *ATP1B2* gene encoding the β_2_ subunit of the Na^+^/K^+^-ATPase holoenzyme (*ATP1B2*:c.130_131insLT796559.1:g.50_276). The SINE insertion caused aberrant RNA splicing. Immunohistochemistry suggested a reduction of ATP1B2 protein expression in the central nervous system of affected puppies. *Atp1b2* knockout mice had previously been reported to show clinical and neurohistopathological findings similar to the affected Malinois puppies. Therefore, we consider *ATP1B2*:c.130_131ins227 the most likely candidate causative variant for a second subtype of SDCA in Malinois dogs, which we propose to term spongy degeneration with cerebellar ataxia subtype 2 (SDCA2). Our study further elucidates the genetic and phenotypic complexity underlying cerebellar dysfunction in Malinois dogs and provides the basis for a genetic test to eradicate one specific neurodegenerative disease from the breeding population in Malinois and the other varieties of the Belgian Shepherd breed. *ATP1B2* thus represents another candidate gene for human inherited cerebellar ataxias, and SDCA2-affected Malinois puppies may serve as a naturally occurring animal model for this disorder.

Inherited (cerebellar) ataxia in humans represents a broad group of clinically, pathologically, and genetically heterogeneous neurodegenerative disorders characterized by progressive degeneration of cerebellum and, to a variable degree, of extracerebellar structures (Manto and Marmolino 2009; Hersheson *et al.* 2012; Jayadev and Bird 2013). Inherited ataxias are divided in autosomal recessive, autosomal dominant, X-linked, and mitochondrial ataxias. Autosomal recessive and autosomal dominant inheritance patterns represent the most prevalent inherited ataxias with ∼20 and 40 identified causative genetic variants so far, respectively (Washington University Neuromuscular Disease Center Web site: http://neuromuscular.wustl.edu; Mancuso *et al.* 2014; Klockgether and Paulson 2011; Jayadev and Bird 2013). Cerebellar ataxia, the main clinical feature of these disorders, becomes manifest as imbalance and lack of coordination. Ataxia may be the sole sign of cerebellar dysfunction or, more frequently, be accompanied by a wide spectrum of additional neurological manifestations (Hersheson *et al.* 2012; Mancuso *et al.* 2014). Disparate pathophysiological mechanisms have been described for inherited cerebellar ataxias, however some recurrent components emerge. These include accumulation of protein aggregates, impaired ion channel functions, defects in the DNA-repair system, and mitochondrial dysfunction (Manto and Marmolino 2009; Klockgether and Paulson 2011; Sandford and Burmeister 2014).

In veterinary medicine, similar to human medicine, cerebellar ataxias are described as a heterogeneous group of neurodegenerative disorders with variability in disease onset, severity and histopathological lesions (Urkasemsin *et al.* 2010; Urkasemsin and Olby 2014). However, to date, a genetic basis has been described for only some autosomal recessive inherited cerebellar ataxias in the dog (Supplemental Material, Table S1, Online Mendelian Inheritance in Animals Web site: http://omia.angis.org.au). Currently, there are no consensus criteria for the classification of canine neurodegenerative diseases, and denominations of entities are mainly based on clinical and/or neuropathological features. The increasing knowledge of the genetic defects underlying these disorders is expected to facilitate the implementation of a neurodegeneration classification scheme in animals and the study of pathogenetic mechanisms (Urkasemsin and Olby 2014).

We studied ataxias in the Belgian Shepherd breed and recently reported a candidate causative variant in the *KCNJ10* gene for spongy degeneration with cerebellar ataxia, subtype 1 (SDCA1). This study revealed an unexpected genetic heterogeneity in clinically comparable cases, suggesting that more than one type of cerebellar ataxia is present in Belgian Shepherd dogs (Kleiter *et al.* 2011; Mauri *et al.* 2017). The *KCNJ10* variant was also identified in an independent study (Stee *et al.* 2016; Van Poucke *et al.* 2017).

The aim of the present study was to identify the presumed causative genetic defect of a second form of SDCA in Belgian Shepherd dogs, which we propose to term SDCA2.

## Materials and Methods

### Ethics statement

All animal experiments were performed according to the local regulations. All dogs in this study were examined with the consent of their owners. The collection of blood samples was approved by the Cantonal Committee for Animal Experiments (Canton of Bern; permit 75/16).

### Breed nomenclature

The Federation Cynologique Internationale (FCI) describes the Malinois, together with the Groenendael, the Laekenois, and the Tervueren, as a variety of the Belgian Shepherd dog breed. The American Kennel Club, however, officially recognizes the Belgian Malinois, the Belgian Sheepdog (FCI: Groenendael), the Belgian Laekenois (FCI: Laekenois), and the Belgian Tervuren (FCI: Tervueren) as four distinct breeds. In this paper all references to the breed nomenclature correspond to the FCI standards.

### Animal selection

**Figure 1:**
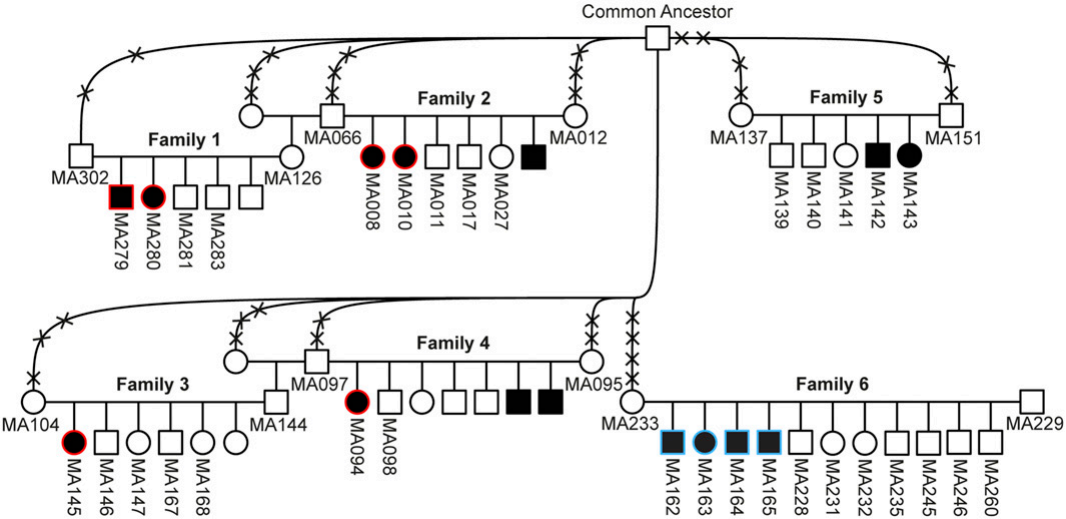
Pedigree of Malinois dogs used for genetic mapping of the disease loci, modified from Mauri *et al.* (2017). Filled symbols represent animals with cerebellar dysfunction. Numbers indicate dogs from which samples were available. Six dogs affected by SDCA1 (*KCNJ10*:c.986T>C) are indicated by red contours. Four affected siblings from family 6, which did not carry the previously identified *KCNJ10* variant, are indicated by blue contours and were selected for homozygosity mapping in this study. The affected animals MA142 and MA143 from family 5 seem to have yet another genetic form of cerebellar dysfunction (see *Results* and *Discussion*). Crosses intersecting the connection lines to the common ancestor represent the numbers of generations (*e.g.*, MA302 is a great-grandson of the common ancestor).

We used the same animals as in our previous study (Mauri *et al.* 2017). We investigated six related Malinois families in which 12 puppies showed clinical signs of cerebellar dysfunction (Figure 1). We also examined seven Malinois puppies with reported cerebellar signs, for which no relatives were available. In addition to these individuals, we genotyped 230 other Malinois, 25 Groenendael, two Laekenois, and 35 Tervueren dogs whose blood samples were donated to the biobank of the Institute of Genetics at the University of Bern. Furthermore, we analyzed 503 samples from 87 genetically diverse dog breeds.

### Neuropathology and immunohistochemistry

Two Malinois puppies from family 5 and three from family 6 with signs of cerebellar dysfunction were necropsied (MA142, MA143, MA162, MA164, MA165, Figure 1). Brain and spinal cord samples from these five puppies were collected and fixed in 4% buffered formaldehyde solution, embedded in paraffin, and sectioned at 2–5 µm. Eye samples were available from puppy MA162 and processed in the same manner. Sections were stained with hematoxylin and eosin and examined by light microscopy. Furthermore, we performed immunohistochemistry (IHC) with a polyclonal rabbit antibody raised against a peptide corresponding to amino acids 115–141 of the human ATP1B2 protein. This epitope is 100% identical between human and dog. IHC was performed on paraffin-embedded cerebellar and brain stem sections from the SDCA2-affected puppies MA162, MA164, MA165; and 13 control dogs, which consisted of nine dogs that did not suffer from cerebellar ataxia and four Malinois puppies that were affected by SDCA1 and homozygous for the *KCNJ10:*c.986T>C variant. To this end, sections were deparaffinized, and antigen heat retrieval was performed by boiling sections in pH 9 buffer (Dako Target Retrieval Solution, pH 9) in a laboratory microwave (20 min at 95°) following a peroxidase block and a blocking step with 10% normal goat serum. Tissue sections were incubated overnight at 4° with the primary antibodies (Thermo Fisher Scientific PA5-26279, 1:50 dilution of the 0.5 mg/ml stock solution) and the reaction was visualized with the Dako REAL Detection System according to the manufacturer’s instructions. The manufacturer’s documentation for the primary antibody showed a Western Blot in which only a single specific band of ∼34 kDa was detected.

### DNA extraction and genotyping

Genomic DNA was isolated from EDTA blood samples with the Maxwell RSC Whole Blood DNA Kit, which were used with the Maxwell RSC Instrument (Promega). Genotyping was done on Illumina CanineHD Chips containing 173,662 genome-wide SNPs by GeneSeek/Neogen. Genotypes were stored in a BC/Gene database version 3.5 (BC/Platforms).

### Linkage and homozygosity mapping

Linkage analysis was performed with Illumina CanineHD SNP Chip genotypes from 20 dogs belonging to family 5 and 6 (Figure 1). We analyzed the data set for parametric linkage under a fully penetrant, recessive model of inheritance with the Merlin software (Abecasis *et al.* 2002). PLINK v1.07 (Purcell *et al.* 2007) was used as described (Wiedmer *et al.* 2016) to search for extended intervals of homozygosity with shared alleles across selected affected animals.

### Reference sequences

The dog CanFam 3.1 genome assembly and NCBI annotation release 103 was used for all analyses. All references to the canine *ATP1B2* gene correspond to the accessions XM_546597.5 (mRNA) and XP_546597.2 (protein). XP_546597.2 has the same length as the human protein (NP_001669.3; 290 amino acids) and 286 out of 290 amino acids (99%) are identical between dog and human.

### Whole-genome resequencing

A PCR-free fragment library was prepared from one affected Malinois dog (MA163) with a 400 bp insert size. We sequenced the library to roughly 32× coverage on an Illumina HiSeq3000 instrument using 2 × 150 bp paired-end reads. The reads were mapped to the dog reference genome assembly CanFam3.1 as previously described (Mauri *et al.* 2017). The sequence data were deposited under the study accession number PRJEB16012 at the European Nucleotide Archive (ENA). The sample accession number is SAMEA104032048. We also used 146 control genomes, which were either publicly available (Bai *et al.* 2015) or produced during other projects in our group (Table S2).

Single nucleotide and small indel variants were individually identified using GATK HaplotypeCaller in gVCF mode, and subsequently genotyped per chromosome and genotyped across all samples simultaneously (Van der Auwera *et al.* 2013). We filtered the obtained data with the variant filtration module of GATK and used the ENSEMBL annotation CanFam 3.1 (version 72) to predict the functional effects of the called variants together with SnpEff software (Cingolani *et al.* 2012). The resulting sequence alignments of MA163 were visually inspected and screened for structural variants with the Integrated Genomics Viewer (IGV) software (Robinson *et al.* 2011).

### PCR and Sanger sequencing

Sanger sequencing was used to confirm the variant identified from whole-genome resequencing. For these experiments we amplified PCR products from genomic DNA using AmpliTaq Gold 360 Master Mix (Life Technologies). The PCR primers used for the genotyping of the *ATP1B2*:c.130_131insLT796559.1:g.50_276 variant were GAACCCCCTGACTCCATTTC (forward primer) and GGAGCAGTTAAAGGCTGGTG (reverse primer). PCR products were directly sequenced on an ABI 3730 capillary sequencer (Life Technologies) after treatment with exonuclease I and shrimp alkaline phosphatase. Sanger sequence data were analyzed with Sequencher 5.1 (GeneCodes). The sequence of the PCR product with the insertion allele was submitted to the ENA under accession number LT796559.1.

### Fragment length analysis

To genotype a large number of samples we used fragment length analyses by assessing the PCR product sizes on the Fragment Analyzer Automated CE (capillary gel electrophoresis) System [Advanced Analytical Technologies (AATI)]. We used the PROSize analytical software (AATI) to visually inspect the obtained gel images and classify the dogs as homozygous for the SINE (short interspersed nuclear elements) insertion (ins/ins, single band of ∼425 bp), heterozygous (wt/ins, two bands of ∼198 and ∼425 bp), or homozygous wild type (wt/wt, single band of ∼198 bp).

### RNA isolation and reverse transcription-PCR

Total RNA was purified from the skin of one affected Malinois puppy (MA133) with the RNeasy Fibrous Tissue Mini Kit according to the manufacturer’s recommendations (QIAGEN). Blood total RNA from one control dog (SY045) was isolated as described (Wiedmer *et al.* 2016). The RNA samples were treated with RNase-free DNase to remove contamination with genomic DNA. The SuperScript IV Reverse Transcriptase kit was used to generate cDNA according to the manufacturer’s recommendations (Thermo Fisher Scientific). Primers for reverse transcription PCR (RT-PCR) were designed in exon 1 and at the boundary of exons 3 and 4 of the *ATP1B2* gene (forward primer: GTGGTTGAGGAGTGGAAGGA; reverse primer: TGGAATCGTTGTAAGGCTCCAA). 30 cycles of PCR were performed with AmpliTaq Gold 360 Master Mix (Life Technologies). RT-PCR products were analyzed with the Fragment Analyzer and preparatively separated using the DNA Size Selection System PippinHT (Sage Science) according to the manufacturer’s recommendations. The resulting isolated fragments were sequenced separately as described above.

### Data availability

File S1 is a video showing the clinical phenotype of the four affected Malinois siblings belonging to family 6 at 4 wk of age (MA162–165). File S2 depicts the sequence context of the 227 bp SINE insertion into exon 2 of the *ATP1B2* gene. File S3 illustrates the aberrant *ATP1B2* exon 2 splicing patterns in dogs with the SINE insertion and the predicted protein sequences resulting from the mutant transcripts. Figure S1 contains a screenshot of the IGV software in the region of the visually identified *ATP1B2*:c.130_131ins227 variant. Table S1 lists the genetic basis of inherited canine cerebellar disorders which have been reported in the literature. Table S2 shows the ENA accession numbers of the whole-genome sequencing data that was used. Table S3 contains genome regions ≥1 Mb that showed positive LOD scores in the linkage analysis. Table S4 presents the homozygous genome regions with shared alleles among the four analyzed affected Malinois puppies from family 6. Table S5 illustrates *ATP1B2*:c.130_131ins227 genotypes of 503 control dogs from 87 diverse dog breeds.

## Results

### Clinical presentation

Clinical signs in the puppies from family 5 and 6 had a similar time of onset of 4 wk of age and were mainly associated with cerebellar dysfunction. We predominantly observed generalized ataxic gait in all puppies. Due to the inability to ambulate, five out of six affected puppies were euthanized on welfare grounds by the sixth week of age. One puppy from family 6 died at 6.5 wk of age during a seizure.

Upon more detailed investigation, only the four affected Malinois puppies from family 6, but not the affected puppies from family 5, additionally had seizures, showed pacing as well as circling, and were diagnosed with central blindness. Moreover, they had a very rapid progression of clinical signs (File S1).

### Neuropathological findings

**Figure 2:**
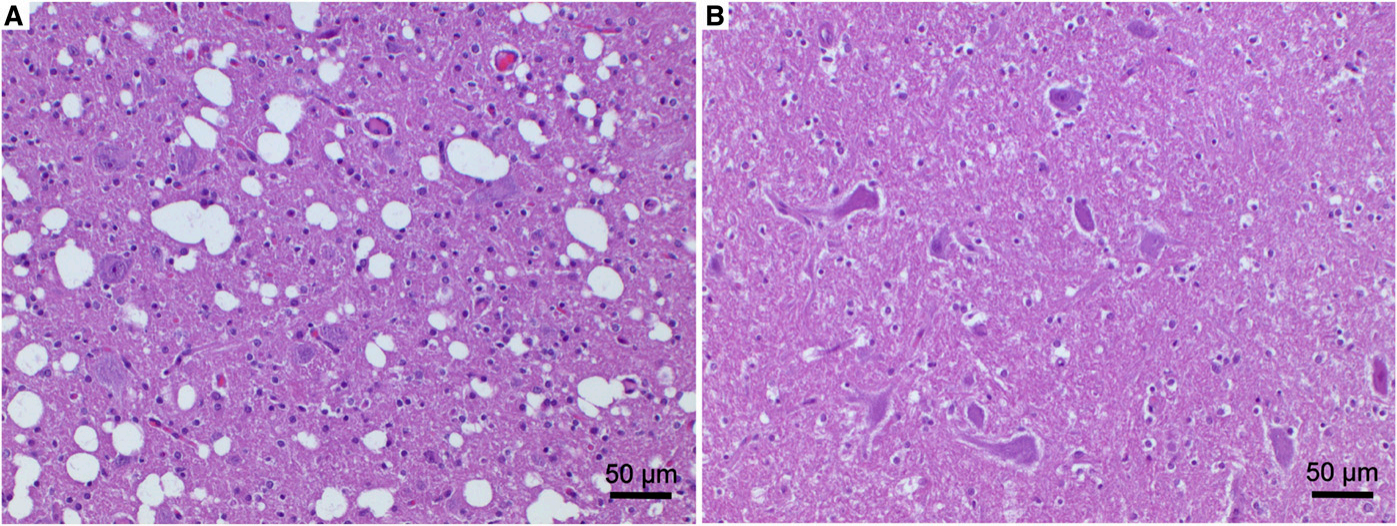
Histopathology of a cerebellar nucleus. (A) Malinois dog MA162 with spongy degeneration and (B) nonaffected control dog. The affected Malinois puppy (A) showed a prominent vacuolation of the neuropil with large numbers of clearly defined and empty vacuoles of varying size and gliosis. Hematoxylin and eosin stain.

Neuropathological analysis was hindered in two puppies due to autolysis of the CNS (MA164, MA165). Histopathological changes in the three affected puppies from family 6 were characterized by bilateral-symmetric vacuolation of the neuropil, targeting the cerebellar nuclei (Figure 2); the ventral horn gray matter of the spinal cord, in particular at the level of the cervical intumescence; and the brain stem. In the spinal cord, vacuolation was associated with neuronal necrosis and severe gliosis. Additionally, in the puppy MA162, neuronal necrosis and diffuse presence of hypertrophic astrocytes with vesicular nuclei, reminiscent of Alzheimer type II cells, were observed in the hippocampus, caudate nucleus, and diffusely in the cortex. Histopathological eye abnormalities were not noticed.

Neuropathological lesions noted in family 5 differed from those observed in family 6. The affected puppies in family 5 displayed gliosis in the cerebellar nuclei, in selected medullary nuclei, and in the spinal cord gray matter. However, CNS vacuolation was not present.

### Genetic mapping of the causative variant in family 6

As the genetic analyses were performed independently and at the same time as the clinical and neuropathological investigations, we initially considered the possibility that the affected puppies in families 5 and 6 shared the same genetic defect. To map the causative locus we investigated these two Malinois families with a total of six puppies with cerebellar dysfunction (Figure 1). A combined linkage analysis revealed a single linked segment on chromosome 5 reaching a statistically significant LOD score of 3.657. However, when we performed linkage analysis separately for each of the two families, we noted that family 6 only presented a single linked segment on chromosome 5 (LOD score of 2.680), whereas family 5 showed linked segments on 12 different chromosomes (maximal LOD score of 0.977, Table S3).

To fine map the region of interest, we then analyzed the six affected Malinois puppies belonging to family 5 and 6 for extended regions of homozygosity with simultaneous allele sharing. This initial homozygosity-mapping approach did not reveal any shared segment on chromosome 5 between all six investigated puppies. As the histopathological findings and the clinical presentation had already suggested phenotypic differences between these two families, we then subsequently performed the homozygosity mapping for each family separately. The analyses showed that only the four affected puppies from family 6 had a homozygous genome region on chromosome 5 (Table S4). By intersecting the linked segment and the homozygous interval from the four cases of family 6, we could define an exact critical interval of 10,564,105 bp at Chr5:29,906,132–40,470,236. Moreover, upon inspection of the SNP-chip genotypes of the isolated Malinois cases with unknown relationships to our families, we identified one additional puppy, which also carried the disease-associated haplotype in the homozygous state (MA133). We therefore assumed that this puppy was affected by the same genetic disease as the four cases in family 6.

### Identification of the causative variant

A total of 255 genes were annotated in the 10.6 Mb critical interval on chromosome 5. To acquire a comprehensive overview of all variants in this region we resequenced the whole genome of one affected Malinois puppy (MA163) and called single nucleotide as well as indel variants with respect to the reference genome of a presumably nonaffected Boxer. The genotype of the affected Malinois puppy was further compared with 146 dog genomes from various breeds that had been sequenced in the course of other studies (Table S2). We hypothesized that the causative variant should be completely absent from all dog breeds in the sample set except the Belgian Shepherd breed. After applying this filter, 37 disease-associated variants remained. However, none of these variants was predicted to change an encoded protein by our automated bioinformatic analysis (Table 1).

**Table 1:** Variants detected by whole-genome resequencing of one affected Malinois puppy (MA163)

Filtering Step	Number of Variants
Variants in the whole genome*^a^*	1,889,727
Variants in the critical 10.6 Mb interval on chromosome 5	75,231
Variants in the critical interval that were absent from 146 other dog genomes	37
Protein-changing variants in the whole genome*^a^*	7936
Protein-changing variants in the 10.6 Mb critical interval on chromosome 5	817
Protein-changing variants in the critical interval, absent from 146 other dog genomes	0

a The sequences were compared to the reference genome (CanFam 3.1) from a Boxer. Protein-changing variants were classified based on the ENSEMBL annotation (version 72).

**Figure 3:**
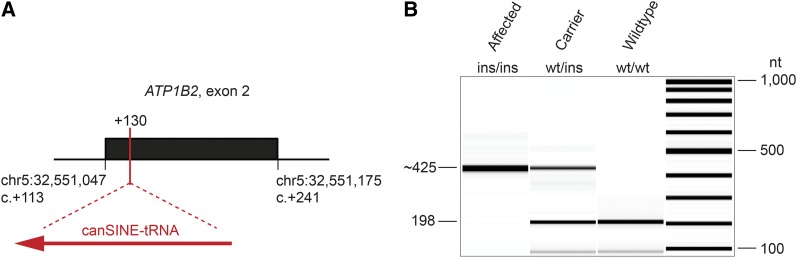
SINE insertion in exon 2 of the *ATP1B2* gene (*ATP1B2*:c.130_131ins227). (A) A 227 bp SINE insertion was found in homozygous state in five Malinois puppies affected by SDCA2 after position 130 of the *ATP1B2* coding sequence. The SINE belonged to the SINEC2A1_CF family derived from an endogenous tRNA gene. 15 nt flanking the insertion site were duplicated. (B) Experimental genotyping of the SINE insertion by fragment size analysis. We amplified exon 2 of *ATP1B2* and flanking intron segments by PCR and separated the products of dogs with the three different genotypes by capillary gel electrophoresis.

Therefore, we considered the possibility that the disease-associated variant was a structural variant, which would not have been detected by our automated variant calling pipeline. Thus, we visually inspected the 10.6 Mb critical interval on chromosome 5 and identified a candidate structural variant in the *ATP1B2* gene (Figure S1). The structural variant arose from a 227 bp SINE insertion within exon 2 of the *ATP1B2* gene, including a 15 bp duplication flanking the insertion site, and can be described as *ATP1B2*:c.130_131insLT796559.1:g.50_276 or in abbreviated form as *ATP1B2*:c.130_131ins227 or Chr5:32,551,064_32,551,065ins227 (Figure 3 and Figure S1).

The presence of this structural variant in homozygous state was confirmed by Sanger sequencing in the four affected Malinois puppies belonging to family 6 and in the isolated case (MA133). The two available parents (MA229, MA233) were heterozygous for this insertion as expected for obligate carriers. Furthermore, we genotyped this variant by fragment length analysis in 251 other Malinois, 25 Groenendael, two Laekenois, 35 Tervueren, and 503 dogs of genetically diverse other breeds. The variant was not found outside the Belgian Shepherd population, but it also occurred in a heterozygous state in 38 Malinois, one Groenendael, and seven Tervueren dogs. In the two cases from family 5 and in the five remaining isolated cases, the variant was also absent (Table 2 and Table S5).

**Table 2:** Association of the SINE insertion with cerebellar dysfunction

Genotype *ATP1B2*:c.130_131ins227	wt/wt	wt/ins	ins/ins
Malinois cases (family 6 and MA133)	—	—	5
Malinois cases (families 1–5 and six isolated puppies)*^a^*	13	1	—
Malinois controls	199	38	—
Groenendael controls	24	1	—
Laekenois controls	2	—	—
Tervueren controls	28	7	—
Control dogs from other breeds*^b^*	503	—	—

a Six of these Malinois puppies, which belonged to family 1–4, and one isolated case, MA152, were previously reported to be affected by SDCA1 caused by the *KCNJ10*:c.986T>C variant (Mauri *et al.* 2017).

b These dogs were specifically genotyped by fragment length analysis for the *ATP1B2*:c.130_131ins227 variant. The genome sequences of 146 independent control dogs were also homozygous wt/wt. Therefore, the number of control dogs totals 948.

### Analyses of the ATP1B2 transcript

**Figure 4:**
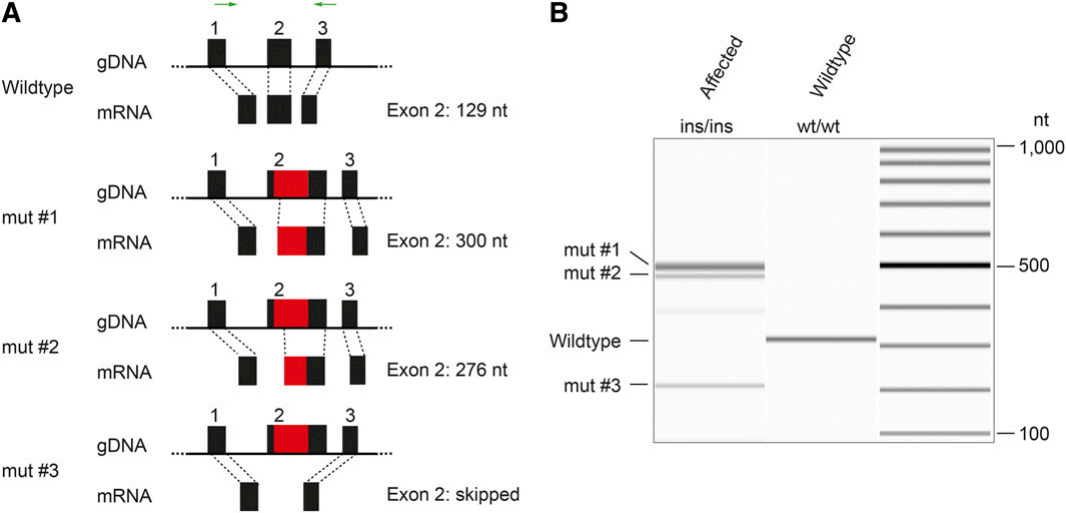
Effect of the SINE insertion on *ATP1B2* transcripts. (A) Schematic representation of exons 1–3 of the *ATP1B2* gene. The 227 bp SINE insertion in exon 2 is displayed in red. Different mutant transcripts were identified and three of them characterized (mut #1–3). In mut #1 and #2 the SINE insertion leads to the utilization of novel splice acceptors, in which parts of the normal exon 2 were replaced with mutant sequences. In mut #3, exon 2 was skipped. RT-PCR primers in exon 1 and at the boundary of exons 3 and 4 of the *ATP1B2* gene are indicated with green arrows (B) RT-PCR products of an affected and a control dog. All products were sequenced to confirm their identities (File S3).

Unfortunately, no suitable brain RNA samples were available for this study. To examine the effect of the 227 bp SINE insertion in exon 2 of the *ATP1B2* gene on the transcript, we therefore isolated total skin and blood RNA from one affected Malinois puppy (MA133) and an unaffected control dog (SY045), respectively. In the affected Malinois puppy we identified at least three distinct transcript isoforms due to altered splicing. After separation and sequencing, none of the experimentally obtained RT-PCR products had the expected size. Sequencing of the products demonstrated either the complete skipping of exon 2 or the activation of two new cryptic splice sites generating aberrant exon lengths. All three mutant transcripts maintained the original reading frame (Figure 4 and File S3).

### IHC

**Figure 5:**
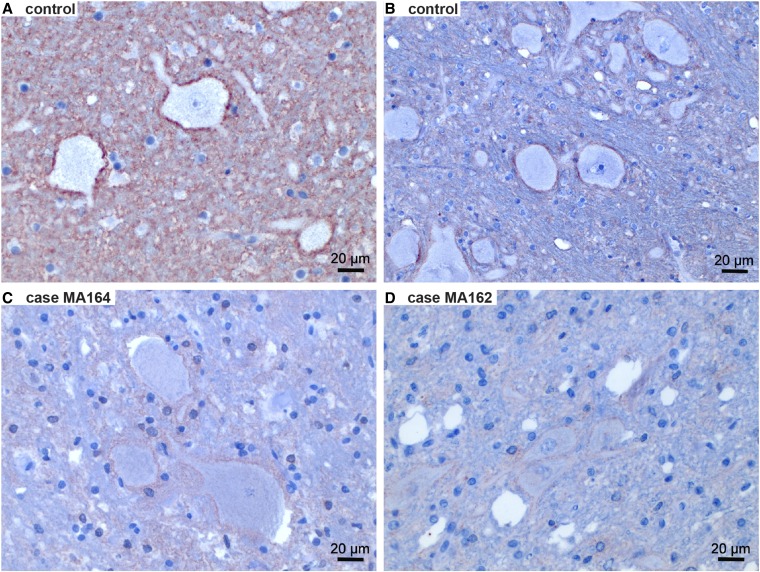
IHC for the ATP2B1 protein. (A and B) Two control dogs showed a clear perineuronal expression of ATP2B1. However, the intensity of the IHC signal was variable between dogs. (C and D) In two of the affected Malinois puppies, perineuronal expression was present, but appeared to be weaker than in the control dogs. In a third affected puppy, no ATP2B1 expression was observed.

To assess the ATP1B2 protein expression we performed IHC on cerebellar and brain stem tissues with anti-ATP1B2 polyclonal antibodies (Figure 5). In the control dogs, the generated IHC signal was consistently present, albeit with varying intensity. The signal was mostly seen around neurons and in a glial-like pattern, with extensions similar to astrocytic processes, in the whole cerebellar and brain-stem parenchyma, especially in the gray matter. The obtained IHC sections from MA162 and MA164 showed a weaker IHC signal around neurons (Figure 5). In the affected Malinois puppy MA165, no ATP2B1 expression could be detected by IHC.

## Discussion

In this study, we identified a structural variant in exon 2 of the *ATP1B2* gene as candidate causative genetic variant for SDCA in the Belgian Shepherd breed. While we only observed affected puppies in the Malinois variety, the proposed pathogenic variant also segregates in other varieties of the Belgian Shepherd breed. We suggest to call this particular phenotype spongy degeneration with cerebellar ataxia, subtype 2 (SDCA2).

To the best of our knowledge, so far, no *ATP1B2* variants have been described in human patients with neurologic symptoms. However, *Atp1b2* knockout mice were reported with similar clinical signs as the affected Malinois puppies. *Atp1b2^−/−^* mice showed rapidly worsening motor impairment and spongy degeneration of the CNS, resembling the neurohistopathological findings observed in the Malinois puppies but with a different topographical distribution (Magyar *et al.* 1994). Our hypothesis is also supported by a study where the Na^+^/K^+^-ATPase was inhibited *in vivo* by subdural injections of ouabain in guinea pigs. The inhibition of the Na^+^/K^+^-ATPase holoenzyme caused seizures and, on histopathology, an evident spongy degeneration in the CNS (Calandriello *et al.* 1995).

The Na^+^/K^+^-ATPase is a ubiquitously expressed multi-subunit protein in the plasma membrane. It is the principal regulator of intracellular homeostasis in every animal cell. The holoenzyme consists of α, β, and auxiliary γ subunits. It is responsible for the active Na^+^ extrusion (three ions) and K^+^ uptake (two ions) necessary to generate and maintain the cellular transmembrane ionic gradients that are essential for the activity of secondary transporters such as voltage-gated Na^+^ and K^+^ channels, the Na^+^/Ca^2+^ exchanger, and neurotransmitter uptake transporters (Mobasheri *et al.* 2000; Tokhtaeva *et al.* 2009; Friedrich, *et al.* 2016). The α subunit is the main component of the Na^+^/K^+^-ATPase and is also defined as a catalytic subunit. It is responsible for ion transport. The β subunit is essential for correct folding, assembly, and targeting of the holoenzyme to the plasma membrane as well as for holoenzyme function by regulating Na^+^ affinity (Habiba *et al.* 2000; Mobasheri *et al.* 2000; Geering 2008; Tokhtaeva *et al.* 2009). Furthermore the β subunit may play a role in cell adhesion and CNS development (Antonicek *et al.* 1987; Müller-Husmann *et al.* 1993; Lecuona *et al.* 1996; Boer *et al.* 2010). In contrast to the α and β subunits, the γ subunit is not essential for the function of the Na^+^/K^+^-ATPase. If present, it acts as an ion transport regulator. To date, four different α, four β, and seven γ subunit isoforms were identified (Geering 2001; Hilbers *et al.* 2016).

The *ATP1B2* gene encodes the β_2_ subunit isoform, first discovered as adhesion molecule on glia (AMOG) (Antonicek *et al.* 1987; Gloor *et al.* 1990). The β_2_ subunit isoform is predominantly expressed in the brain, especially in the cerebellum, and it preferentially binds to α_2_, which is mainly found in astrocytes after completion of development. The main task of the α_2_β_2_ Na^+^/K^+^-ATPase holoenzyme in astrocytes is to restore extracellular K^+^ homeostasis following neuronal depolarization. A failure of K^+^ buffering and clearance would result in high extracellular K^+^ and consequently to sustained glial and neuronal hyperexcitability compromising neuronal firing, synaptic transmission, and neurotransmitter reuptake (Tokhtaeva *et al.* 2012; Friedrich *et al.* 2016; Hilbers *et al.* 2016; Larsen *et al.* 2016).

Disturbances in the K^+^ homeostasis in the CNS are often associated with neurological disorders such as cerebellar dysfunction or epilepsy (Hirose 2006; Larsen *et al.* 2014). Recently, we genetically characterized a hereditary cerebellar ataxia in the Belgian Shepherd breed caused by a pathologic variant in *KCNJ10*, encoding the astrocytic K_ir_4.1 potassium channel (Mauri *et al.* 2017). Both KCNJ10 and the α_2_β_2_ Na^+^/K^+^-ATPase seem to play a pivotal role for K^+^ homeostasis in the CNS, especially in the cerebellum. They are not interchangeable, but serve temporally distinct roles, with KCNJ10 acting during and α_2_β_2_ Na^+^/K^+^-ATPase working after neuronal depolarization (Larsen *et al.* 2014). In addition to an impaired K^+^ clearance after neuronal depolarization, dysfunction of the α_2_β_2_ Na^+^/K^+^-ATPase might also lead to extracellular accumulation of glutamate and/or to an increase in intracellular Ca^2+^ and/or to cell swelling, which all might contribute to the observed histopathological changes (Friedrich *et al.* 2016; Prontera *et al.* 2017).

The central blindness in the SDCA2-affected puppies is most likely explained by necrotic changes involving the visual cortex. However, the α_3_β_2_ Na^+^/K^+^-ATPase holoenzyme is also associated with retinoschisin on the photoreceptor and bipolar cells of the eye, and the retinoschisin–α_3_β_2_ Na^+^/K^+^-ATPase complex is necessary for maintenance of retinal cell organization as well as photoreceptor-bipolar synaptic structure. Variants in the retinoschisin gene, *RS1*, cause splitting of retinal cell layers and loss in central vision. The phenotype is defined as X-linked juvenile retinoschisis (XLRS) (MIM#312700; Molday *et al.* 2007, 2012; Friedrich *et al.* 2011). However, we did not observe any histopathological changes of the retina in an SDCA2-affected puppy and thus think that blindness in these dogs is caused by the central lesions.

Our RNA experiments demonstrated altered splicing of the *ATB1B2* transcript in skin RNA from one affected puppy. It has to be cautioned that the splicing might be different in the CNS. However, given the sequence context of the SINE insertion, it seems impossible that the insertion allele could give rise to the expression of the wild-type mRNA.

Our IHC findings suggest a reduction in ATP1B2 protein expression in the CNS of SDCA2-affected Malinois. However, IHC signal could still be identified, possibly indicating the expression of mutant ATP1B2 protein. The antibody that was used was directed against a peptide corresponding to amino acids 115–141 located in the extracellular domain of the wild-type protein. The three characterized aberrant transcripts all maintained the original reading frame and encoded this epitope. Thus, translation of any of these mutant transcripts might have led to the expression of a detectable mutant protein (File S3). Further investigation is needed to assess if any of these mutant ATP1B2 proteins might retain some residual functional activity. For the mut #3 protein, this seems highly unlikely as it is lacking the entire predicted transmembrane domain. The mut #1 and mut #2 proteins contain insertions of 59 and 51 amino acids into the cytoplasmic domain respectively, and it is at least questionable whether they can exert the same functions as the normal β_2_ subunit of the Na^+^/K^+^-ATPase.

In humans, *ATP1B2* variants have not been reported to date. However, severe neurological disorders have been associated with variants in the *ATP1A2* and *ATP1A3* genes encoding the α_2_ and α_3_ subunit isoforms. *ATP1A2* variants are responsible for hemiplegic migraine type 2 (FHM2, MIM#602481; Prontera *et al.* 2017) and alternating hemiplegia of childhood 1 (AHC1, MIM#104290; Swoboda *et al.* 2004). *ATP1A3* defects cause rapid-onset dystonia Parkinsonism (RDP, DYT12, MIM#128235), as well as alternating hemiplegia of childhood 2 (AHC2, MIM#614820) and CAPOS syndrome (cerebellar ataxia, areflexia, pes cavus, optic atrophy, and sensorineural hearing loss syndrome, MIM#601338; Heinzen *et al.* 2014; Holm and Lykke-Hartmann 2016).

To conclude, we identified a SINE insertion in exon 2 of the *ATP1B2* gene (*ATP1B2*:c.130_131insLT796559.1:g.50_276), leading to altered splicing and an impaired ATP1B2 protein expression in the CNS, as most likely causative for SDCA2 in the Belgian Shepherd breed. Cerebellar dysfunction in this breed is heterogeneous and, together with the previously reported *KCNJ10*:c.986T>C variant, we still cannot explain all cases affected by similar clinical signs. Further investigation is needed to resolve the genetic and phenotypic complexity underlying cerebellar dysfunction in Malinois dogs. Our findings encourage genetic testing of Belgian Shepherd dogs so that the nonintentional breeding of affected puppies with SDCA2 can be avoided in the future. Moreover, our data imply *ATP1B2* as an additional candidate gene for human inherited cerebellar ataxias of unknown etiology. Affected puppies represent a spontaneous animal model for hereditary ataxia.

## Supplementary Material

Supplemental material is available online at www.g3journal.org/lookup/suppl/doi:10.1534/g3.117.043018/-/DC1.
