# Supplementary material for: A SINE Insertion in ATP1B2 in Belgian Shepherd Dogs Affected by Spongy Degeneration with Cerebellar Ataxia (SDCA2)
Source: G3 (Bethesda). 2017 Jun 15;7(8):2729–37. doi: 10.1534/g3.117.043018 (PMC5555477; doi:10.1534/g3.117.043018)
Supplement: Supplementary file 2 [file 2729FileS2.pdf]

File S2. Sequence context of the 227 bp SINE insertion into exon 2 of the canine *ATP1B2* gene

Genetic variant:	Chr5:32,551,064_32,551,065ins227	(CanFam 3.1 assembly)
	ATP1B2:c.130_131ins227	(XM_546597.5)
	ATP1B2:c.130_131insLT796559.1:g.50_276	(XM_546597.5)

CanFam 3.1, chromosome 5 genomic sequence, positions 32,550,985 - 32,551,224 (NC_006587.3)

Wildtype *ATP1B2* exon 2, 129 nucleotides in black CAPITAL LETTERS (129 nt)

227 bp SINE-insertion in blue

PCR primers for the amplification of a genomic 198 bp product (wildtype allele) vs. a genomic 425 bp product (variant allele) are indicated in green and beneath the sequence with “>>>” and “<<<”.

```
32,551,001      atcacagggg acctggaacc ccctgactcc atttcctcct ccctagCCTT CATCCTCCTC      32,551,060
32,551,061      TTCT            >>>>>>>>>>>>>>>>>>>>>>                                 32,551,064
                15 nt duplication flanking the insertion

32,551,064.1    TTTTTTTTTT TTTTTTTTTT TTTTTTTTTT TTTTAAAGAT TTTATTTATT TATTCATGAT      32,551,064.60
32,551,064.61   AGTCACAGAG AGAGAGAGAG GCAGAGACAC AGGCAGAGGG AGAAGCAGGC TCCACGCACT      32,551,064.120
32,551,064.121  GGGAGCCCGA TGTGGGATTC GATCCCGGGT CTCCAGGATC GCGCCCTGGG CCAAAGGCAG      32,551,064.180
32,551,064.181  GCGCCAAACC GCTGCACCAC CCAGGGATCC CCTCATCCTC CTCTTCT                      32,551,064.227
                                                   15 nt duplication flanking the insertion

32,551,065          ACCTCG TCTTCTATGG CTTCCTCACG GCTATGTTCA CCCTCACCAT GTGGGTCATG      32,551,120
32,551,121      CTGCAGACCG TGTCTGACCA TACCCCCAAG TATCAGGACC GGCTGGCCAC ACCGGgtgag      32,551,180
32,551,181      tgatgcggct ccccaccagc ctttaactgc tcctgtgctg ccaagcctcc ccccaaaata      32,551,240
                              <<<<<<<<<<<<<<<<<<<<<<
```
