# Supplementary material for: A SINE Insertion in ATP1B2 in Belgian Shepherd Dogs Affected by Spongy Degeneration with Cerebellar Ataxia (SDCA2)
Source: G3 (Bethesda). 2017 Jun 15;7(8):2729–37. doi: 10.1534/g3.117.043018 (PMC5555477; doi:10.1534/g3.117.043018)
Supplement: Supplementary file 3 [file 2729FileS3.pdf]

File S3. Wildtype and mutant *ATP1B2* exon 2 splicing patterns and predicted protein isoforms

Genetic variant:	Chr5:32,551,064_32,551,065ins227	(CanFam 3.1 assembly)
	ATP1B2:c.130_131ins227	(XM_546597.5)
	ATP1B2:c.130_131insLT796559.1:g.50_276	(XM_546597.5)

(A) Exon 2 splicing patterns

CanFam 3.1, chromosome 5 genomic sequence (NC_006587.3).

Wildtype *ATP1B2* exon 2, 129 nucleotides in black CAPITAL LETTERS (129 nt)

SINE-insertion in blue CAPITAL LETTERS

Bases retained in the transcript are highlighted in grey

Wildtype (without SINE insertion)

32,551,001	atcacagggg acctggaacc ccctgactcc atttcctcct ccct**ag**CCTT CATCCTCCTC	32,551,060
32,551,061	TTCTACCTCG TCTTCTATGG CTTCCTCACG GCTATGTTCA CCCTCACCAT GTGGGTCATG	32,551,120
32,551,121	CTGCAGACCG TGTCTGACCA TACCCCCAAG TATCAGGACC GGCTGGCCAC ACCGGgtgag	32,551,180

mut #1

32,551,001	atcacagggg acctggaacc ccctgactcc atttcctcct ccctagCCTT CATCCTCCTC	32,551,060
32,551,061	TTCT	32,551,064
32,551,064.1	TTTTTTTTTT TTTTTTTTTT TTTTTTTTTT TTTTAA**AG**AT TTTATTTATT TATTCATGAT	32,551,064.60
32,551,064.61	AGTCACAGAG AGAGAGAGAG GCAGAGACAC AGGCAGAGGG AGAAGCAGGC TCCACGCACT	32,551,064.120
32,551,064.121	GGGAGCCCGA TGTGGGATTC GATCCCGGGT CTCCAGGATC GCGCCCTGGG CCAAAGGCAG	32,551,064.180
32,551,064.181	GCGCCAAACC GCTGCACCAC CCAGGGATCC CCTCATCCTC CTCTTCT	32,551,064.227
32,551,065	ACCTCG TCTTCTATGG CTTCCTCACG GCTATGTTCA CCCTCACCAT GTGGGTCATG	32,551,120
32,551,121	CTGCAGACCG TGTCTGACCA TACCCCCAAG TATCAGGACC GGCTGGCCAC ACCGGgtgag	32,551,180

mut #2

32,551,001	atcacagggg acctggaacc ccctgactcc atttcctcct ccctagCCTT CATCCTCCTC	32,551,060
32,551,061	TTCT	32,551,064
32,551,064.1	TTTTTTTTTT TTTTTTTTTT TTTTTTTTTT TTTTAAAGAT TTTATTTATT TATTCATGAT	32,551,064.60
32,551,064.61	**AG**TCACAGAG AGAGAGAGAG GCAGAGACAC AGGCAGAGGG AGAAGCAGGC TCCACGCACT	32,551,064.120
32,551,064.121	GGGAGCCCGA TGTGGGATTC GATCCCGGGT CTCCAGGATC GCGCCCTGGG CCAAAGGCAG	32,551,064.180
32,551,064.181	GCGCCAAACC GCTGCACCAC CCAGGGATCC CCTCATCCTC CTCTTCT	32,551,064.227
32,551,065	ACCTCG TCTTCTATGG CTTCCTCACG GCTATGTTCA CCCTCACCAT GTGGGTCATG	32,551,120
32,551,121	CTGCAGACCG TGTCTGACCA TACCCCCAAG TATCAGGACC GGCTGGCCAC ACCGGgtgag	32,551,180

mut #3

(Exon 2 skipped entirely)

32,551,001	atcacagggg acctggaacc ccctgactcc atttcctcct ccctagCCTT CATCCTCCTC	32,551,060
32,551,061	TTCT	32,551,064
32,551,064.1	TTTTTTTTTT TTTTTTTTTT TTTTTTTTTT TTTTAAAGAT TTTATTTATT TATTCATGAT	32,551,064.60
32,551,064.61	AGTCACAGAG AGAGAGAGAG GCAGAGACAC AGGCAGAGGG AGAAGCAGGC TCCACGCACT	32,551,064.120
32,551,064.121	GGGAGCCCGA TGTGGGATTC GATCCCGGGT CTCCAGGATC GCGCCCTGGG CCAAAGGCAG	32,551,064.180
32,551,064.181	GCGCCAAACC GCTGCACCAC CCAGGGATCC CCTCATCCTC CTCTTCT	32,551,064.227
32,551,065	ACCTCG TCTTCTATGG CTTCCTCACG GCTATGTTCA CCCTCACCAT GTGGGTCATG	32,551,120
32,551,121	CTGCAGACCG TGTCTGACCA TACCCCCAAG TATCAGGACC GGCTGGCCAC ACCGGgtgag	32,551,180

(B) Predicted translated protein sequences

For each sequence the translation of the entire coding sequence is given. The predicted transmembrane domain is highlighted in grey. In the wildtype ATP1B2 protein amino acids 1-39 are predicted to be cytoplasmic and amino acids 68-290 are predicted to constitute the extracellular domain (Uniprot accession P14415, human ATP1B2). The peptide sequence, which was used to generate the anti-ATP1B2 antibodies corresponds to amino acids 115-141 of the wildtype protein. This sequence is underlined in all the proteins shown below.

Wildtype (translated from wildtype allele without SINE insertion); corresponds to XP_546597.2)

(Amino acids 38 - 80 encoded by exon 2 are shown in green.)

```
  1  MVIQKEKKSC GQVVEEWKEF VWNPRTHQFM GRTGTSWAFI LLFYLVFYGF LTAMFTLTMW     60
 61  VMLQTVSDHT PKYQDRLATP GLMIRPKTEN LDVIVNVSDT ESWDQHVQKL NKFLEPYNDS    120
121  IQAQKNDVCR PGRYYEQPDN GVLNYPKRAC QFNRTQLGDC SGIGDPTHYG YSTGQPCVFI    180
181  KMNRVINFYA GANQSMNVTC VGKRDEDAEN LGNFVMFPAN GNIDLMYFPY YGKRFHVNYT    240
241  QPLVAVKFLN VTPNVEVNVE CRINAANIAT DDERDKFAGR VAFKLRINKT               290
```

mut #1

(Aberrant amino acids 38-96 encoded by the SINE insertion are shown in blue, amino acids 97 – 137, which correspond to regular residues encoded by the wildtype exon 2 are shown in green.)

```
  1  MVIQKEKKSC GQVVEEWKEF VWNPRTHQFM GRTGTSWDFI YLFMIVTERE RGRDTGRGRS     60
 61  RLHALGARCG IRSRVSRIAP WAKGRRQTAA PPRDPLILLF YLVFYGFLTA MFTLTMWVML    120
121  QTVSDHTPKY QDRLATPGLM IRPKTENLDV IVNVSDTESW DQHVQKLNKF LEPYNDSIQA    180
181  QKNDVCRPGR YYEQPDNGVL NYPKRACQFN RTQLGDCSGI GDPTHYGYST GQPCVFIKMN    240
241  RVINFYAGAN QSMNVTCVGK RDEDAENLGN FVMFPANGNI DLMYFPYYGK RFHVNYTQPL    300
301  VAVKFLNVTP NVEVNVECRI NAANIATDDE RDKFAGRVAF KLRINKT                  347
```

mut #2

(Aberrant amino acids 38-88 encoded by the SINE insertion are shown in blue, amino acids 89 – 129, which correspond to regular residues encoded by the wildtype exon 2 are shown in green.)

```
  1  MVIQKEKKSC GQVVEEWKEF VWNPRTHQFM GRTGTSWVTE RERGRDTGRG RSRLHALGAR     60
 61  CGIRSRVSRI APWAKGRRQT AAPPRDPLIL LFYLVFYGFL TAMFTLTMWV MLQTVSDHTP    120
121  KYQDRLATPG LMIRPKTENL DVIVNVSDTE SWDQHVQKLN KFLEPYNDSI QAQKNDVCRP    180
181  GRYYEQPDNG VLNYPKRACQ FNRTQLGDCS GIGDPTHYGY STGQPCVFIK MNRVINFYAG    240
241  ANQSMNVTCV GKRDEDAENL GNFVMFPANG NIDLMYFPYY GKRFHVNYTQ PLVAVKFLNV    300
301  TPNVEVNVEC RINAANIATD DERDKFAGRV AFKLRINKT                           339
```

mut #3

(This predicted protein lacks amino acids 38-80 including the transmembrane domain of the wildtype sequence.)

```
  1  MVIQKEKKSC GQVVEEWKEF VWNPRTHQFM GRTGTSWGLM IRPKTENLDV IVNVSDTESW     60
 61  DQHVQKLNKF LEPYNDSIQA QKNDVCRPGR YYEQPDNGVL NYPKRACQFN RTQLGDCSGI    120
121  GDPTHYGYST GQPCVFIKMN RVINFYAGAN QSMNVTCVGK RDEDAENLGN FVMFPANGNI    180
181  DLMYFPYYGK RFHVNYTQPL VAVKFLNVTP NVEVNVECRI NAANIATDDE RDKFAGRVAF    240
241  KLRINKT                                                              247
```
