# Supplementary material for: A SINE Insertion in ATP1B2 in Belgian Shepherd Dogs Affected by Spongy Degeneration with Cerebellar Ataxia (SDCA2)
Source: G3 (Bethesda). 2017 Jun 15;7(8):2729–37. doi: 10.1534/g3.117.043018 (PMC5555477; doi:10.1534/g3.117.043018)
Supplement: Supplementary file 4 [file 2729FigureS1.pdf]

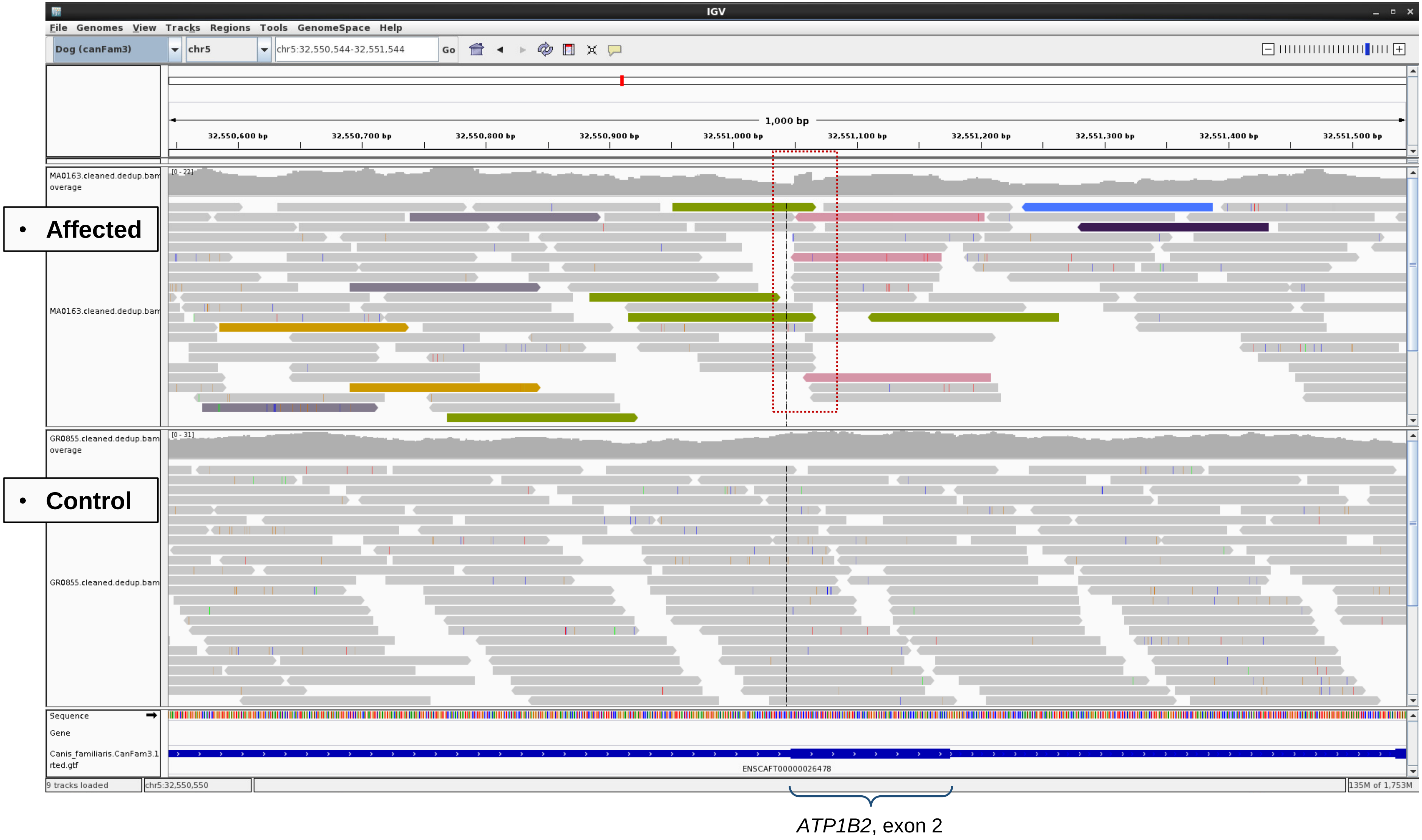

Figure S1. IGV screenshot of the region with the 227 nt SINE insertion within exon 2 of the *ATP1B2* gene (*ATP1B2*:c.130_131insLT796559.1:g.50_276). The sequence alignments of the affected Malinois (MA163) and a Golden Retriever control dog (GR0855) are displayed. The dashed red box indicates a region in the affected dog where forward and reverse read alignments are truncated. The truncated reads overlap by 15 duplicated nucleotides, flanking the insertion site of the SINE. The control dog shows normal overlapping alignments of the reads with respect to the reference sequence. Both libraries were sequenced on an Illumina HiSeq 3000 instrument using 2x150 bp paired-end reads.
