# Supplementary material for: A SINE Insertion in ATP1B2 in Belgian Shepherd Dogs Affected by Spongy Degeneration with Cerebellar Ataxia (SDCA2)
Source: G3 (Bethesda). 2017 Jun 15;7(8):2729–37. doi: 10.1534/g3.117.043018 (PMC5555477; doi:10.1534/g3.117.043018)
Supplement: Supplementary file 5 [file 2729TableS1.pdf]

Table S1. Inherited cerebellar ataxias in the dog with known genetic basis described to date

Gene Symbol	Gene Full Name	Protein Function	Affected Dog breed(s)	Phenotype name in Dog(s)	OMIA	Phenotype name in Human	Common Feature 1	Common Feature 2
ATG4D	Autophagy related 4D cysteine peptidase	Component of the autophagy-lysosome pathway	Lagotto Romagnolo	Neurodegenerative vacuolar storage disease (Kyöstilä *et al.* 2015)	001954-9615	-	Protein degradation pathway	
RAB24	RAB24, member RAS oncogene family	Described as necessary for autophagy, which allows global turnover of cellular organelles as well as clearance of potentially toxic long-lived proteins and allows intracellular neuronal homeostasis	Old English Sheepdogs & Gordon Setters	Canine hereditary ataxia (Agler *et al.* 2014)	001913-9615	-		
SEL1L	SEL1L ERAD E3 ligase adaptor subunit	Component of the endoplasmic reticulum associated protein degradation machinery that targets misfolded proteins to the ubiquitin proteasome system for degradation	Finnish Hound	Canine progressive early-onset cerebellar ataxia (Kyöstilä *et al.* 2012)	001692-9615	-		
SNX14	Sorting nexin 14	Essential role in neural development and function, particularly in the autophagy degradation pathway	Hungarian Vizsla	Cerebellar cortical degeneration (Fenn *et al.* 2016)	002034-9615	SCAR20 (MIM#616354) (Thomas *et al.* 2014; Shukla *et al.* 2017)		Neural development
CAPN1	Calpain 1	The exact role of this protease in humans is not clear to date. It has been suggested to be important for synaptic plasticity and development of the CNS	Parson Russell Terrier	Spinocerebellar Ataxia (Forman *et al.* 2013)	001820-9615	SPG76 (MIM#616907) (Gan-Or *et al.* 2016; Wang *et al.* 2016)	Possible involvement in protein degradation pathway	
SPTBN2	Spectrin beta, non-erythrocytic 2 (β-III spectrin)	Critical for Purkinje cells development. Stabilize the glutamate transporter EAAT4 of Purkinje cells, facilitate protein trafficking, maintain high density of sodium channels	Beagle	Neonatal cerebellar cortical degeneration (Forman *et al.* 2012)	002092-9615	SCA5 (MIM#600224) & SCAR14 (MIM#615386) (Jackson *et al.* 2001; Ikeda *et al.* 2006; Elsayed *et al.* 2014)		
VLDLR	Very low density lipoprotein receptor	Essential in regulating the correct cortical and cerebellar neuronal migration during embryonic development	Eurasier	Dandy-Walker-like malformation (M. Gerber *et al.* 2015)	001947-9615	CAMRQ1 (MIM# 224050) (Boycott *et al.* 2005; Micalizzi *et al.* 2016)		
GRM1	Glutamate metabotropic receptor 1 (mGluR1)	Essential role in cerebellar development and synaptic plasticity by modulating intracellular Ca^{2+} levels and neuronal excitability	Coton de Tulear	Bandera's neonatal ataxia (Zeng *et al.* 2011)	000078-9615	SCAR13 (MIM#614831) (Guergueltcheva *et al.* 2012)	Ion channel	
ITPR1	Inositol 1,4,5-trisphosphate receptor type 1 (IP$_3$R1)	Central role in the regulation of intracellular Ca^{2+} concentration. Involvement of Ca^{2+} in developmental events and gene expression has been suggested	Italian Spinone	Spinocerebellar ataxia (Forman *et al.* 2015)	002097-9615	SCA15 (MIM#606658) & SCA29 (MIM#117360) & GLSP (MIM#206700) (Huang *et al.* 2012; Tada *et al.* 2016; S. Gerber *et al.* 2016)		
KCNJ10	Potassium voltage-gated channel subfamily J member 10 (K$_{ir}$4.1)	Key role in modulating the resting membrane potential of neuronal cells through potassium spatial buffering	Belgian Shepherd<br><br>Several Russell Terriers	SDCA1 (Mauri *et al.* 2017; Van Poucke *et al.* 2017)<br>SAMS (Gilliam *et al.* 2014)	002089-9615	EAST Syndrome (MIM#612780) (Bockenhauer *et al.* 2009; Abdelhadi *et al.* 2016)		

Abbreviations: SCAR: autosomal recessive spinocerebellar ataxia; CNS: central nervous system; CAMRQ1: autosomal recessive cerebellar hypoplasia and mental retardation with or without quadrupedal locomotion 1; SPG76: autosomal recessive spastic paraplegia 76; GLSP: Gillespie Syndrome; SDCA1: spongy degeneration with cerebellar ataxia 1; SAMS: spinocerebellar ataxia with myokymia, seizures, or both; EAST: epilepsy, ataxia, sensorineural deafness, and renal salt wasting tubulopathy.

LITERATURE CITED (in Table S1)

Abdelhadi, O., D. Iancu, H. Stanescu, R. Kleta, and D. Bockenhauer, 2016 EAST Syndrome: Clinical, Pathophysiological, and Genetic Aspects of Mutations in KCNJ10. Rare Dis. 4: e1195043.

Agler, C., D. M. Nielsen, G. Urkasemsin, A. Singleton, N. Tonomura *et al.*, 2014 Canine Hereditary Ataxia in Old English Sheepdogs and Gordon Setters Is Associated with a Defect in the Autophagy Gene Encoding RAB24. PLoS Genet. 10: e1003991.

Bockenhauer, D., S. Feather, H. C. Stanescu, S. Bandulik, A. A. Zdebik *et al.*, 2009 Epilepsy, Ataxia, Sensorineural Deafness, Tubulopathy, and KCNJ10 Mutations. N. Engl. J. Med. 360: 1960–1970.

Boycott, K. M., S. Flavelle, A. Bureau, H. C. Glass, T. M. Fujiwara *et al.*, 2005 Homozygous Deletion of the Very Low Density Lipoprotein Receptor Gene Causes Autosomal Recessive Cerebellar Hypoplasia with Cerebral Gyral Simplification. Am. .J Hum. Genet. 77: 477–483.

Elsayed, S. M., R. Heller, M. Thoenes, M. S. Zaki, D. Swan *et al.*, 2014 Autosomal Dominant SCA5 and Autosomal Recessive Infantile SCA Are Allelic Conditions Resulting from SPTBN2 Mutations. Eur. J. Hum. Genet. 22: 286–288.

Fenn, J., M. Boursnell, R. J. Hitti, C. A. Jenkins, R. L. Terry *et al.*, 2016 Genome Sequencing Reveals a Splice Donor Site Mutation in the SNX14 Gene Associated with a Novel Cerebellar Cortical Degeneration in the Hungarian Vizsla Dog Breed. BMC Genet. 17: 123.

Forman, O. P., L. De Risio, K. Matiasek, S. Platt, and C. Mellersh, 2015 Spinocerebellar Ataxia in the Italian Spinone Dog Is Associated with an Intronic GAA Repeat Expansion in ITPR1. Mamm. Genome 26: 108–117.

Forman, O. P., L. De Risio, and C. S. Mellersh, 2013 Missense Mutation in CAPN1 Is Associated with Spinocerebellar Ataxia in the Parson Russell Terrier Dog Breed. PLoS One 8: e64627.

Forman, O. P., L. De Risio, J. Stewart, C. S. Mellersh, and E. Beltran, 2012 Genome-Wide mRNA Sequencing of a Single Canine Cerebellar Cortical Degeneration Case Leads to the Identification of a Disease Associated SPTBN2 Mutation. BMC Genet. 13: 55.

Gan-Or, Z., N. Bouslam, N. Birouk, A. Lissouba, D. B. Chambers *et al.*, 2016 Mutations in CAPN1 Cause Autosomal-Recessive Hereditary Spastic Paraplegia. Am. J. Hum. Genet. 98: 1038–1046.

Gerber, M., A. Fischer, V. Jagannathan, M. Drögemüller, C. Drögemüller *et al.*, 2015 A Deletion in the VLDLR Gene in Eurasier Dogs with Cerebellar Hypoplasia Resembling a Dandy-Walker-like Malformation (DWLM). PLoS One 10: e0108917.

Gerber, S., K. J. Alzayady, L. Burglen, D. Brémond-Gignac, V. Marchesin *et al.*, 2016 Recessive and Dominant De Novo ITPR1 Mutations Cause Gillespie Syndrome. Am. J. Hum. Genet. 98: 971–980.

Gilliam, D., D.P. O’Brien, J.R. Coates, G.S. Johnson, G.C. Johnson *et al.*, 2014 A Homozygous KCNJ10 Mutation in Jack Russell Terriers and Related Breeds with Spinocerebellar Ataxia with Myokymia, Seizures, or Both. J. Vet. Inter. Med. 28: 871–877.

Guergueltcheva, V., D. N. Azmanov, D. Angelicheva, K. R. Smith, T. Chamova *et al.*, 2012 Autosomal-Recessive Congenital Cerebellar Ataxia Is Caused by Mutations in Metabotropic Glutamate Receptor 1. Am. J. Hum. Genet. 91: 553–564.

Huang, L., J. Warman Chardon, M. T. Carter, K. L. Friend, T. E. Dudding *et al.*, 2012 Missense Mutations in ITPR1 Cause Autosomal Dominant Congenital Nonprogressive Spinocerebellar Ataxia. Orphanet J. Rare Dis. 7: 67.

Ikeda, Y., K. A. Dick, M. R. Weatherspoon, D. Gincel, K. R. Armbrust *et al.*, 2006 Spectrin Mutations Cause Spinocerebellar Ataxia Type 5. Nat. Genet. 38: 184–190.

Jackson, M., W. Song, M. Y. Liu, L. Jin, M. Dykes-Hoberg *et al.*, 2001 Modulation of the Neuronal Glutamate Transporter EAAT4 by Two Interacting Proteins. Nature 410: 89–93.

Kyöstilä, K., S. Cizinauskas, E. H. Seppälä, E. Suhonen, J. Jeserevics *et al.*, 2012 A SEL1L Mutation Links a Canine Progressive Early-Onset Cerebellar Ataxia to the Endoplasmic Reticulum-Associated Protein Degradation (ERAD) Machinery. PLoS Genet. 8: e1002759.

Kyöstilä, K., P. Syrjä, V. Jagannathan, G. Chandrasekar, T. S. Jokinen *et al.*, 2015 A Missense Change in the ATG4D Gene Links Aberrant Autophagy to a Neurodegenerative Vacuolar Storage Disease. PLoS Genet. 11: e1005169.

Mauri, N., M. Kleiter, M. Leschnik, S. Högler, E. Dietschi *et al.*, 2017 A Missense Variant in KCNJ10 in Belgian Shepherd Dogs Affected by Spongy Degeneration with Cerebellar Ataxia (SDCA1). G3 (Bethesda) 7: 663–669.

Micalizzi, A., I. Moroni, M. Ginevrino, T. Biagini, T. Mazza *et al.*, 2016 Very Mild Features of Dysequilibrium Syndrome Associated with a Novel VLDLR Missense Mutation. Neurogenetics 17: 191–195.

Shukla, A., P. Upadhyai, J. Shah, K. Neethukrishna, S. Bielas *et al.*, 2017 Autosomal Recessive Spinocerebellar Ataxia 20: Report of a New Patient and Review of Literature. Eur. J. Med. Genet. 60: 118–123.

Tada, M., M. Nishizawa, and O. Onodera, 2016 Roles of Inositol 1,4,5-Trisphosphate Receptors in Spinocerebellar Ataxias. Neurochem. Int. 94: 1–8.

Thomas, A. C., H. Williams, N. Setó-Salvia, C. Bacchelli, D. Jenkins *et al.*, 2014 Mutations in SNX14 Cause a Distinctive Autosomal-Recessive Cerebellar Ataxia and Intellectual Disability Syndrome. Am. J. Hum. Genet. 95: 611–621.

Van Poucke, M., K. Stee, S. F. M. Bhatti, A. Vanhaesebrouck, L. Bosseler *et al.*, 2017 The Novel Homozygous KCNJ10 c.986T>C (p.(Leu329Pro)) Variant Is Pathogenic for the SeSAME/EAST Homologue in Malinois Dogs. Eur. J. Hum. Genet. 25: 222–226.

Wang, Y., J. Hersheson, D. Lopez, M. Hammer, Y. Liu *et al.*, 2016 Defects in the CAPN1 Gene Result in Alterations in Cerebellar Development and Cerebellar Ataxia in Mice and Humans. Cell Rep. 16: 79–91.

Zeng, R., F. H. G. Farias, G. S. Johnson, S. D. McKay, R. D. Schnabel *et al.*, 2011 A Truncated Retrotransposon Disrupts the GRM1 Coding Sequence in Coton de Tulear Dogs with Bandera's Neonatal Ataxia. J. Vet. Inter. Med. 25: 267–272.
